# Demonstration of indigenous malaria elimination through Track-Test-Treat-Track (T4) strategy in a Malaria Elimination Demonstration Project in Mandla, Madhya Pradesh

**DOI:** 10.1186/s12936-020-03402-6

**Published:** 2020-09-17

**Authors:** Praveen K. Bharti, Harsh Rajvanshi, Sekh Nisar, Himanshu Jayswar, Kalyan B. Saha, Man Mohan Shukla, Ashok K. Mishra, Ravendra K. Sharma, Aparup Das, Harpreet Kaur, Suman L. Wattal, Altaf A. Lal

**Affiliations:** 1 Indian Council of Medical Research - National Institute of Research in Tribal Health (ICMR-NIRTH), Jabalpur, Madhya Pradesh India; 2 Malaria Elimination Demonstration Project, Mandla, Madhya Pradesh India; 3 Government of Madhya Pradesh, Directorate of Health Services, Bhopal, India; 4 Indian Council of Medical Research, Department of Health Research, Ministry of Health and Family Welfare, New Delhi, India; 5 National Vector Borne Disease Control Programme, Ministry of Health and Family Welfare, New Delhi, India; 6 Foundation for Disease Elimination and Control of India, Mumbai, Maharashtra India

## Abstract

**Background:**

Many malaria endemic countries are heading towards malaria elimination through the use of case management and vector control strategies, which employ surveillance, improving access to early diagnosis, prompt treatment., and integrated vector control measures. There is a consensus that elimination of malaria is feasible when rapid detection and prompt treatment is combined with mosquito-human contact interruption in an efficient and sustainable manner at community levels. This paper describes results of an integrated case management and vector control strategy for reducing malaria cases in 1233 villages over 3 years in district Mandla, Madhya Pradesh, India.

**Methods:**

The project enrolled the entire population (1,143,126) of Mandla district for fever surveillance followed by testing of febrile cases and treatment of positive subjects using T4 strategy, which is Track (by fever), Test (by RDTs), Treat (by ACT) and Track (for completion of treatment). In addition to the active and passive surveillance for detection and treatment of febrile cases, the project conducted mass screening and treatment to clear the asymptomatic reservoirs of infection. Febrile cases were also tested in the out-patient department of the District Hospital from June 2018 to September, 2018 and in a community-based medical camp from November 7 to 14, 2019. The project also used vector control measures for interrupting human-mosquito contact, and information, education and communication (IEC) campaigns to increase demand for malaria services at community level.

**Results:**

This project has revealed about 91% reduction of indigenous cases of malaria during the period from June 2017 to May 2020, through case management and vector control strategies. A total 357,143 febrile cases were screened, out of which 0.19% were found positive for the presence of malaria parasites, with *Plasmodium falciparum* and *Plasmodium vivax* ratio of 62:38. The prevalence of malaria was higher in individuals > 15 years of age (69% cases). The positivity rate was 0.33% in 2017–18, 0.13% in 2018–19, and 0.06% in 2019–20. In all of the 3 years of the project, the peak transmission correlated with rains. Mass screening revealed 0.18% positivity in Sep-Oct 2018, followed by 0.06% in June 2019, and 0.03% in December 2019, and these were mostly asymptomatic cases in the community. Imported cases into the district were mostly contributed by the distant state of Telangana (51.13%). Fever patients tested for malaria parasites in the District Hospital and medical camp revealed zero cases.

**Conclusion:**

Using the current intervention and prevention tools along with optimum utilization of human resources, a 91% reduction in indigenous cases of malaria was seen in the district in 3 years. The reduction was similar in the three high prevalence blocks of the district. These results reveal that malaria elimination is achievable in India within a stipulated time frame. The reduction of malaria at the community level was further validated when zero malaria cases were diagnosed during hospital and community-based studies in Mandla. Prompt detection and treatment of imported/migratory cases may have prevented outbreaks in the district. This project has demonstrated that field programmes backed by adequate technical, management, operational, and financial controls with robust monitoring are needed for achieving malaria elimination.

## Background

Malaria in India is a major public health problem in the rural and tribal communities and contributes significantly to the overall malaria burden in South East Asia region [1].

India together with sub-Saharan Africa accounts for 85% of global malaria morbidity and mortality [1]. The World Health Organization (WHO) has set the goal to eliminate malaria from 26 countries including India by the year 2030. The Government of India has moved forward to achieve this goal in the stipulated timeline. The National Vector Borne Disease Control Programme (NVBDCP) has developed the National Strategic Plan (NSP) to eliminate malaria by 2027, 3-years ahead of global target [2].

India recorded significant drop from 75 million cases in 1950s to 0.1 million cases in year 1961, but the efforts could not be sustained. As a consequence, the number of cases increased to 6.45 million cases in year 1976 [3]. This resurgence was attributed to complexities in malaria, unique topography and climatic change, genetic composition of the parasite immune status, drugs resistance, vector diversity and insecticide resistance, asymptomatic malaria, migration malaria, various other operational administrative and technical reasons [4, 5].

The World Malaria Report 2019 has revealed a 49% reduction in malaria cases in India from 2017 to 2018 [1]. When malaria cases in 2018 are compared with 2015, a remarkable reduction of 63% has been found [6]. The percentage of reduction was noticeably high for *Plasmodium falciparum* cases (73%) as compared to *P. vivax* (43%). This reduction was mainly due to reduction in cases reported from the state of Odisha, where a significant reduction (by 85%) was achieved by using Comprehensive Case Management Programme (CCMP) [3].

In Odisha, an initiative to reduce asymptomatic reservoirs from the community was undertaken through the *“Durgam Anchalare* Malaria *Nirakarana*” (DAMaN) project [7]. The CCMP approach for early detection and treatment of malaria along with DAMaN initiative resulted in reducing the size of infectious reservoirs [8]. The strengthening of malaria case management system through community-based intervention has been also implemented in various countries for malaria control [8–12].

Elimination of malaria from India presents unique challenges because of its population, topography, diverse climate, and uneven capacity of health care delivery in rural areas. These challenges contribute to challenges in conducting robust surveillance and obtaining reliable disease burden data. For instance, Hay et al. reported that actual malaria incidence in India could be 09 to 50 times greater than the reported [13] and another study in the same year reported that malaria mortality could be 13-fold higher [14].

With the objective to demonstrate that malaria can be eliminated, and re-introduction can be quickly detected and prevented, the Malaria Elimination Demonstration Project (MEDP) was initiated in the nine blocks of Mandla’s 1233 villages. This project was initiated as a public private partnership between the Government of Madhya Pradesh (GoMP), Indian Council for Medical Research (ICMR) and Foundation for Disease Elimination and Control of India (FDEC-India) (a CSR subsidiary of Sun Pharmaceutical Industries Ltd.) in the tribal district of Mandla district in Madhya Pradesh. The study design of the project is described in companion paper [15]. Other companion papers describe the socio-demographics, entomological, capacity building, IEC/BCC, vector control, digital surveillance aspects of MEDP along with a model for malaria elimination based on learnings from the project.

## Methods

### Description of study area

The project was implemented in all 1233 villages spread across nine blocks of Mandla district in Madhya Pradesh, India. Mandla is a part of the Jabalpur Division in the Mahakoshal region and most of the district lies along the basin of the River Narmada. The district has an area of 8771 km^2^ with over 2,022 km^2^ area covered by deciduous forest. The district receives a moderate amount of rainfall every year and the monsoon season lasts from June to September. The total population of the district is 11,43,126 (as of May 2020) with 87% residing in the rural areas. The male to female ratio is almost equal and literacy is 66.8%. About 58% population belong to Scheduled Tribes, mainly *‘Gond’* and *‘Baiga’* tribes.

The Indoor Residual Spray (IRS) of the vector control measures in the district used alphacypermethrin 5%. The Long-Lasting Insecticidal Nets (LLINs) were distributed in 2017 and 2019. IRS and LLIN distributions were done as part of routine government activity through the District Malaria Office with supervisory support of MEDP staff.

The project used Information Education Communication (IEC) and Behaviour Change Communication (BCC) as a key strategy in the overall plan. The IEC/BCC material consisting of calendars, flipbooks, job-aids, posters, booths etc. was developed using feedback from the community. These activities were performed in middle schools, community markets (*haat bazaars)* and as part of regular door-to-door fever surveillance.

Active Surveillance: Door-to-door active fever surveillance was done by trained Village Malaria Workers (VMWs), who were equipped with diagnostic and treatment kits (RDT, anti-malarial, anti-pyretic and analgesic). A total of 235 VMWs did surveillance, IEC, diagnosis and treatment work in 1233 villages, which is about 6–8 villages per worker. Six to eight VMWs were supervised by one Malaria Field Coordinator (MFC). For active surveillance, a detailed Advance Tour Plan (ATP), was prepared for each worker to conduct active fever surveillance within a stipulated timeline of 7–14 days [15]. In addition to the active surveillance, passive surveillance was conducted through Accredited Social and Health Activists (ASHAs), who are the village-based health staff of the state government and are trained in malaria diagnosis and treatment.

The Village Malaria Workers used the T4 strategy (Track, Test, Treat, and Track), where they tracked and identified the fever case using active surveillance; tested the case using the bivalent Rapid Diagnostic Test (RDT) kit for diagnoses of *P. falciparum* and *P. vivax* malaria; treated the positive cases using anti-malarials; and tracked the case for successful completion of treatment regimen.

All RDT positive cases were treated as per the national drugs policy 2013 [16]. The fever cases that tested negative by were provided antipyretics and advised to visit nearest government health care facilities for further treatment. Pregnant women that tested positive by RDT and serious malaria cases were promptly referred for treatment to the nearest government hospitals and followed-up. Tracking of positive cases from Sentinel Surveillance Sites (SSS) from January 2019 was also done to determine; (1) the number of suspected malaria cases who seek treatment from private practitioners; and (2) whether these individuals receive appropriate diagnostic and treatment support. Structured questionnaires were developed to record information on households, active and passive cases, and mass screenings on paper-based formats till July 2018. Following which, the project transitioned its data processes to a digital mobile surveillance application tool.

### Mass Surveillance and Treatment (MSAT)

To identify and treat the asymptomatic reservoir of infection in the community, Mass Surveillance and Treatment (MSAT) was done in the hard-to-reach areas, high burden areas (> 5 API), moderate burden areas (1–4.99 API), and low burden areas (0–1 API) in 2018. The sample size for MSAT was calculated based on the existing malaria prevalence at 95% confidence interval. A total of 38,248 population was targeted (> 5 API-9842, 1 to 4.99 API-13,246, 0 to 1 API-7981 and hard to reach areas -7197). Subsequently, in June 2019, MSAT was carried out only in high and low malaria endemic areas targeting a sample size of 6200 and 8500 population, respectively. In December 2019 (once the cases were reduced), another round of MSAT was carried out in 50 houses surrounding the cryptic cases diagnosed and 8467 population was screened.

### Passive surveillance

Fever screening was conducted in the Mandla District Hospital from June 2018 to September 2018, which is the peak malaria transmission season. All fever cases that reported to the District Hospital’s Out-Patient Department (OPD) every Monday, which is the busiest day of the week, were tested for malaria using RDT. Another fever screening was carried out at a community medical camp for the district Mandla organized by the Rotary India (Rotary’s Active Hands Are Touching–RAHAT) from during November 7, 2019 to November 14, 2019. All fever cases were tested for malaria using RDT. Private practitioners were enrolled in the sentinel surveillance network and data on OPD cases, proportion of fever cases, and malaria positive cases was recorded.

### Statistical analysis

Data was collected using a modified active fever surveillance tool of the National Vector Borne Disease Control Programme (NVBDCP). The data analysis was done using Statistical Package of Social Sciences (SPSS) v20 by IBM. Measures of disease frequency were calculated as prevalence rates, followed by calculation of odds ratio between different years. The test of significance of proportions was applied to calculate the p value at 95% confidence interval.

## Results

### Active surveillance

**Fig. 1:**
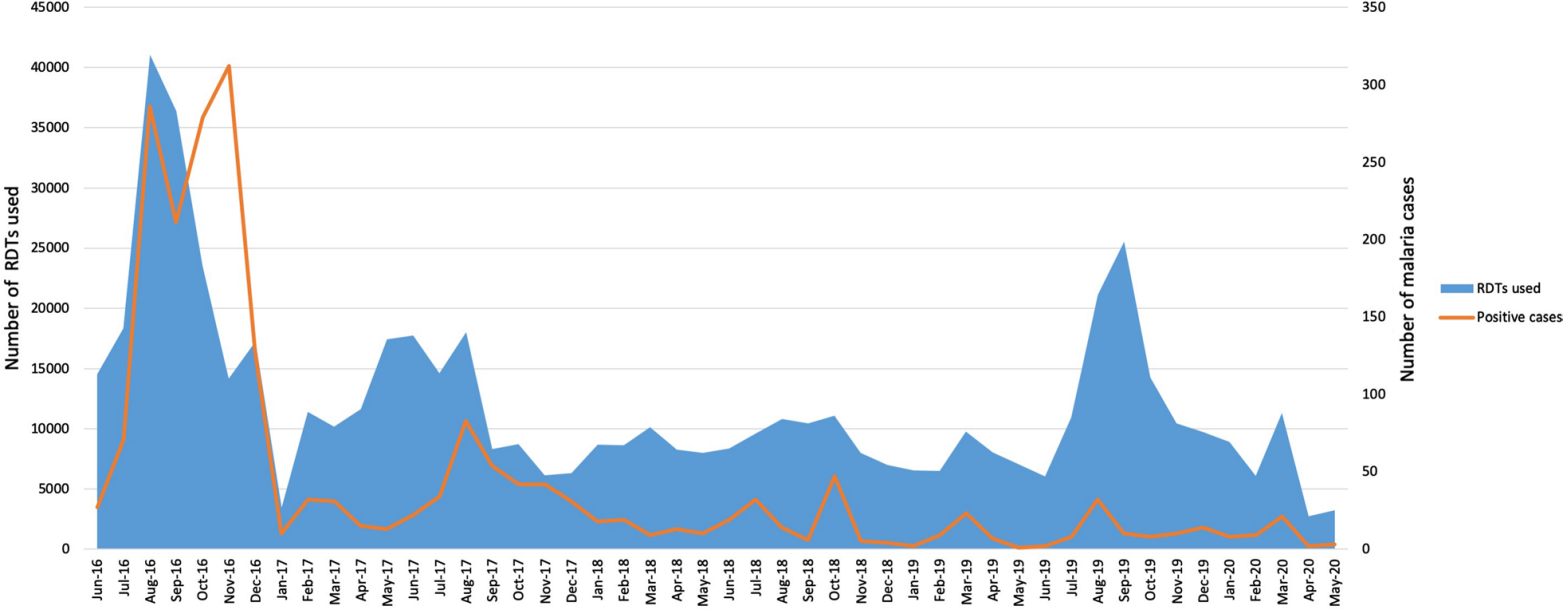
Number of Rapid Diagnostic Test kits (RDTs) tested (357143) from June 2017 to May 2020 (blue area) and the positive cases treated (673) (orange line)

**Fig. 2:**
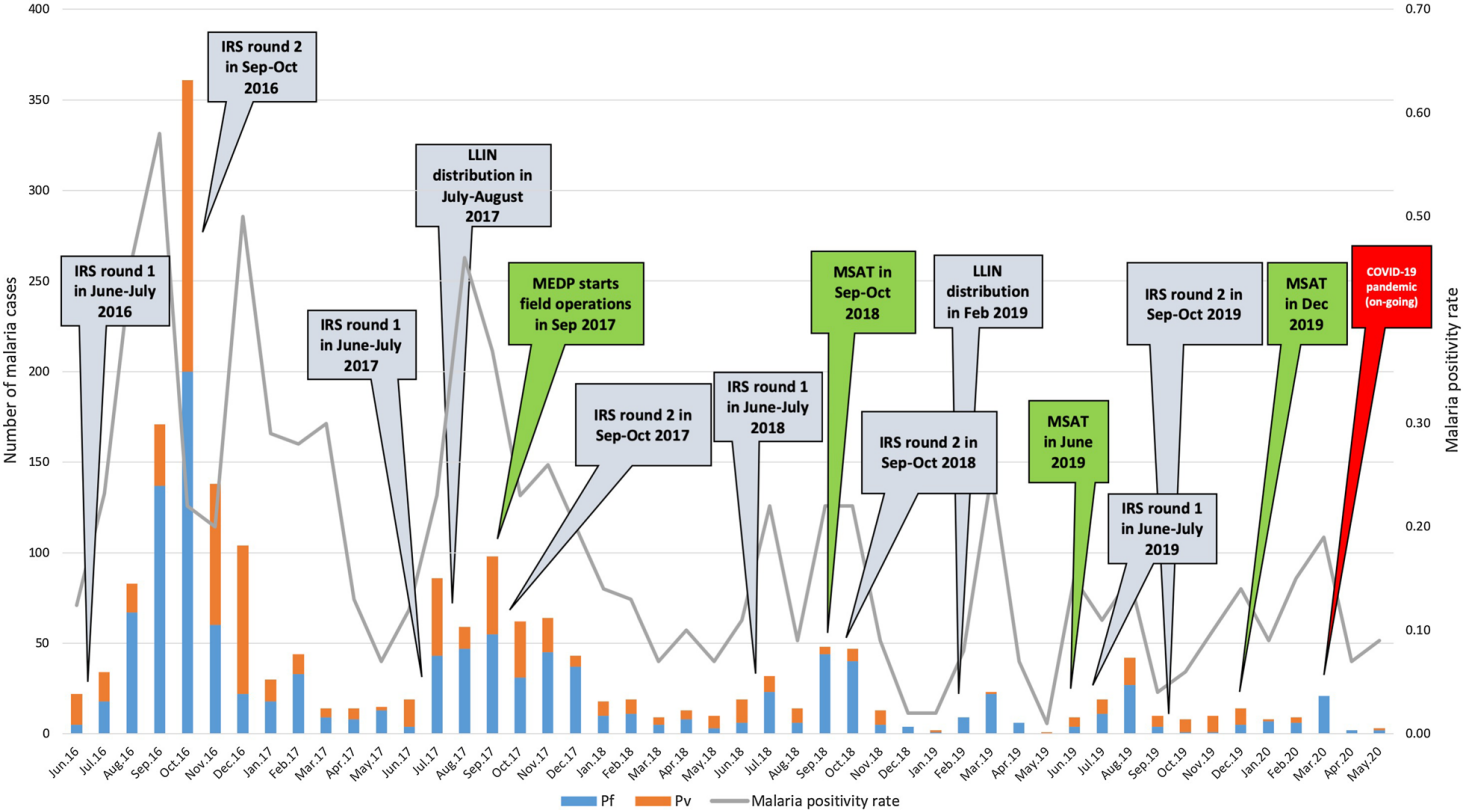
Month-wise and species-wise malaria cases from June 2016 to May 2020 in Mandla district with major events in the timeline. These events include introduction of various vector control interventions, field operations by MEDP, mass screening and treatment

The project started its field operations from September 2017. The data from June 2017 to August 2017 has been provided by the District Malaria Office, Government of Madhya Pradesh to allow analysis of data for consecutive 3 years (from June 2017 to May 2020). During this period, a total of 357,143 febrile cases were screened using RDTs, out of which, 673 (0.19%) tested positive for malaria (Fig. 1), which were promptly treated. The *P. falciparum* and *P. vivax* ratio was 68:32, which indicated higher burden of malaria due to *P. falciparum* as compared to *P. vivax.* Out of 357,143 individuals, 352,891 (98.8%) were tested once, 3988 (1.1%) twice (mean time between two test: 62 ± 40 days) and 264 (0.1%) more than two times (mean time between two tests: 62 ± 40 day) during study duration (Table 1). Age wise analysis revealed that 69% cases were from individuals more than 15 years of age, followed by 21% from five to 15 years, and 10% cases from children under five years of age. The year-wise fever cases and malaria positivity rates along with reduction in cases are shown in Table 2. The month-wise and species-wise malaria prevalence data along with vector control and mass screening interventions are presented in Fig. 2.

**Table 1:** Number of RDTs performed between June to May for 2017–18, 18–19, and 19–20

Period	RDTs done	Fever patients tested one time	No. of positive cases	Fever patients tested two times	No. of positive cases	Mean time between two test ± SD (days)	Fever patients tested more than two times	No. of positive cases	Mean time between more than two tests ± SD (days)
June 17–May 18^a^	123641	121927	313	1606	60	78 ± 70	108	4	63 ± 41
June 18–May 19	103143	101713	118	1340	49	63 ± 35	90	2	43 ± 23
June 19–May 20	130359	129251	105	1043	22	44 ± 14	65	0	31 ± 11
Total	357143	352891	536	3988	131	62 ± 40	264	6	46 ± 25

Number of times the same patient was tested more than once in a year (recurring fever episodes) along with mean difference between the multiple tests have been compared

^a^Project data available from September 2017. Earlier 3 months data (June 2017 to August 2017) is from District Malaria Office, Govt. of MP

**Table 2:** Comparison of malaria status in Mandla from June 2016 to May 2020

Period	No. of test done	No. of malaria cases	Positivity rate	*P. falciparum* %	Malaria prevalence rate (per 100,000)	% reduction	Odds ratio	p value
June 16–May 17	219363	1414	0.64	70.86	123	Base year (reference)	Base year (reference) OR = 1	Base year (reference)
June 17–May 18^a^	123641	377	0.30	60.74	33	73	0.471 (0.421–0.528)	< 0.0001
June 18–May 19	103143	169	0.16	73.96	15	88	0.253 (0.216–0.297)	< 0.0001
June 19–May 20	130359	127	0.10	51.97	11	91	0.150 (0.125–0.180)	< 0.05

Number of tests, cases, positivity rate, Pf %, malaria prevalence, reduction, OR, and significance have been checked from June to May of 2016–17, 17–18, 18–19, and 19–20

^a^Project data available from September 2017. Earlier three months data (June 2017 to August 2017) is from District Malaria Office, Govt. of MP

**Fig. 3:**
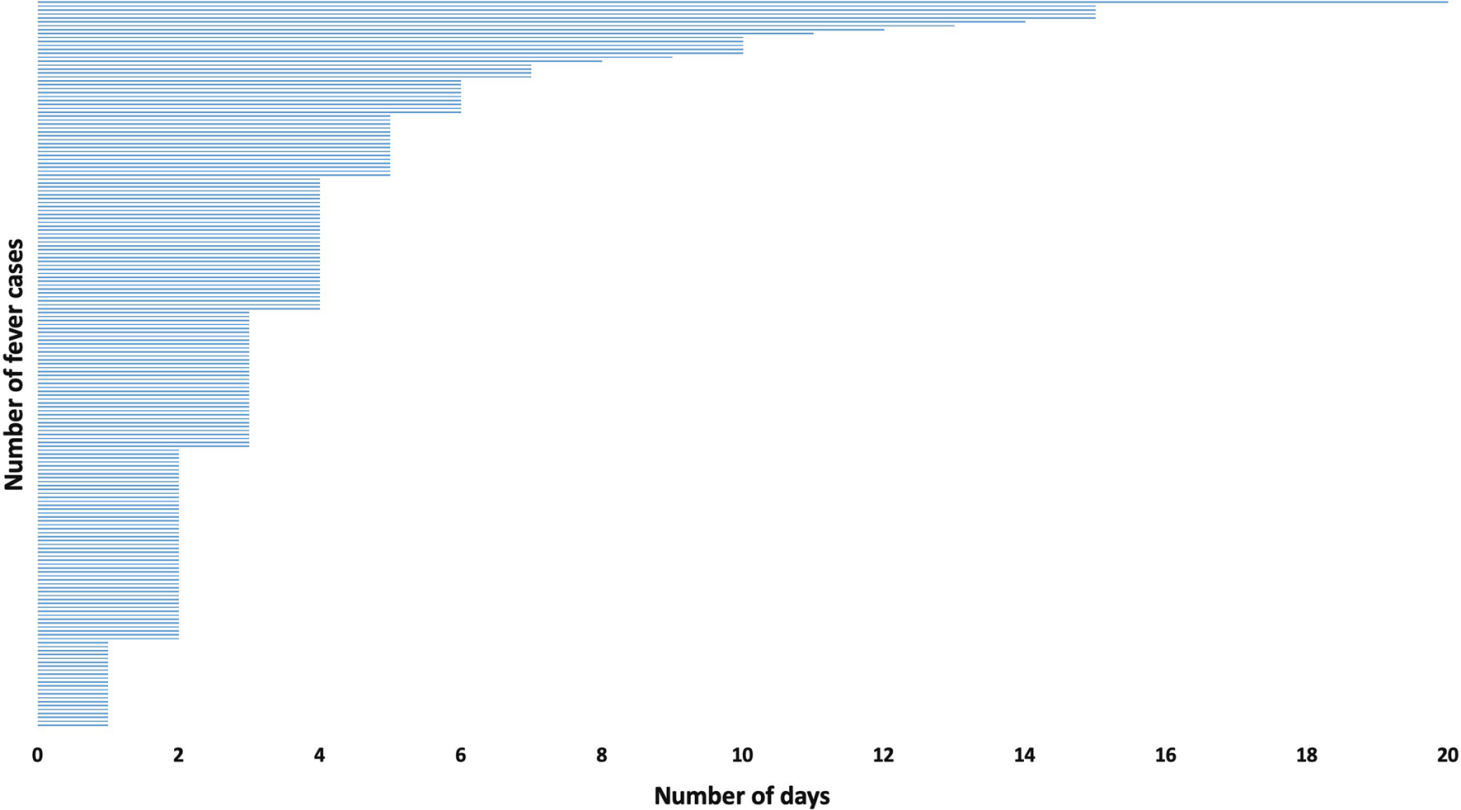
Diagnosis in days from the onset of fever or malaria like illness. Within 48 h of onset of fever, 38% cases were diagnosed and 84% cases were diagnosed within 5 days of onset of fever

Through active surveillance, MEDP identified 38% of febrile cases within 48 h and more than 80% within 5 days of self-reported fever (Fig. 3). Further analysis showed that during the first year (June 2017 to May 2018), the malaria positivity rate of indigenous cases was 0.30 (377/123,641), while in year 2 it was 0.16 (169/103,143), and in year 3 it was 0.01 (127/130,359) (p < 0.001). This 91% reduction was achieved through conventional vector control and case management strategies, with stringent management and operational controls at staff and supply-chain levels and through weekly in-depth reviews. These controls included introduction of Advance Tour Plans (ATP) for staff that conducted field work, real-time data reporting with focus on data quality and integrity, stringent supply chain management systems, weekly reviews, and increased accountability at all levels of the project. Detailed description of these tools and strategies is provided in companion paper and the project website [15, 17–23].

Imported/migratory cases: During routine surveillance in April 2018, six out of the thirteen malaria cases were diagnosed from the village of block, Mohgaon. Following an epidemiological investigation of these cases, it was found that those that tested positive had returned after working in agricultural farms in a distant state of Telangana. After this observation, a comprehensive case information form was developed to determine the geographic nature of infection as per the classification by the Centers of Disease Control and Prevention (CDC) [24] and the WHO [25]. Since then, diagnosis and treatment of 172 imported cases has been done till May 2020. The results shown in Table 3 are very revealing, and inform us that the importation of malaria in Mandla mostly comes from the far-distant place of Telangana state (51.13%), followed by other districts of Madhya Pradesh (22.56%) and Andhra Pradesh (9.02%), adjoining state of Maharashtra (6.77%) and Chhattisgarh (5.26%), and distant states of Uttar Pradesh (1.5%), Karnataka (1.5%), Bihar (0.75%), Gujarat (0.75%) and Rajasthan (0.75%). Almost all imported cases provided history of travel outside Mandla for work (crop harvesting) and may have been infected at place of work.

**Table 3:** Contribution of imported malaria cases into Mandla district from different parts of India along with distance (in Kms) from the project area

State	Contribution %	Distance from Mandla (In Kms)
Telangana	51.13	750
Madhya Pradesh (other districts)	22.56	50–300
Andhra Pradesh	9.02	1035
Maharashtra	6.77	725
Chattisgarh	5.26	270
Uttar Pradesh	1.50	700
Karnataka	1.50	1200
Bihar	0.75	850
Gujarat	0.75	1200
Rajasthan	0.75	1150

In Mandla, the monsoon starts from June and extends till September each year. However, in 2016, the peak was observed in the month of October 2016 with 361 malaria cases. Compared to this, 62 cases in 2017 and 47 cases in 2018 were detected, respectively. Out of 47 cases reported in October 2018, 41 were imported from other states and districts. *P. falciparum* infections were more than *P. vivax,* and mixed infections throughout the study duration (Fig. 2). Out of the nine blocks of Mandla, three blocks contributed maximum cases throughout the study period (Bicchiya, Ghughari, and Mawai). However, there was significant reduction in the number of cases between 2017 and 18 (260 cases), 2018–19 (78 cases), and 2019–20 (26 cases) demonstrating up to 90% reduction in these high-prevalence blocks.

### Mass Screening and Treatment (MSAT)

**Fig. 4:**
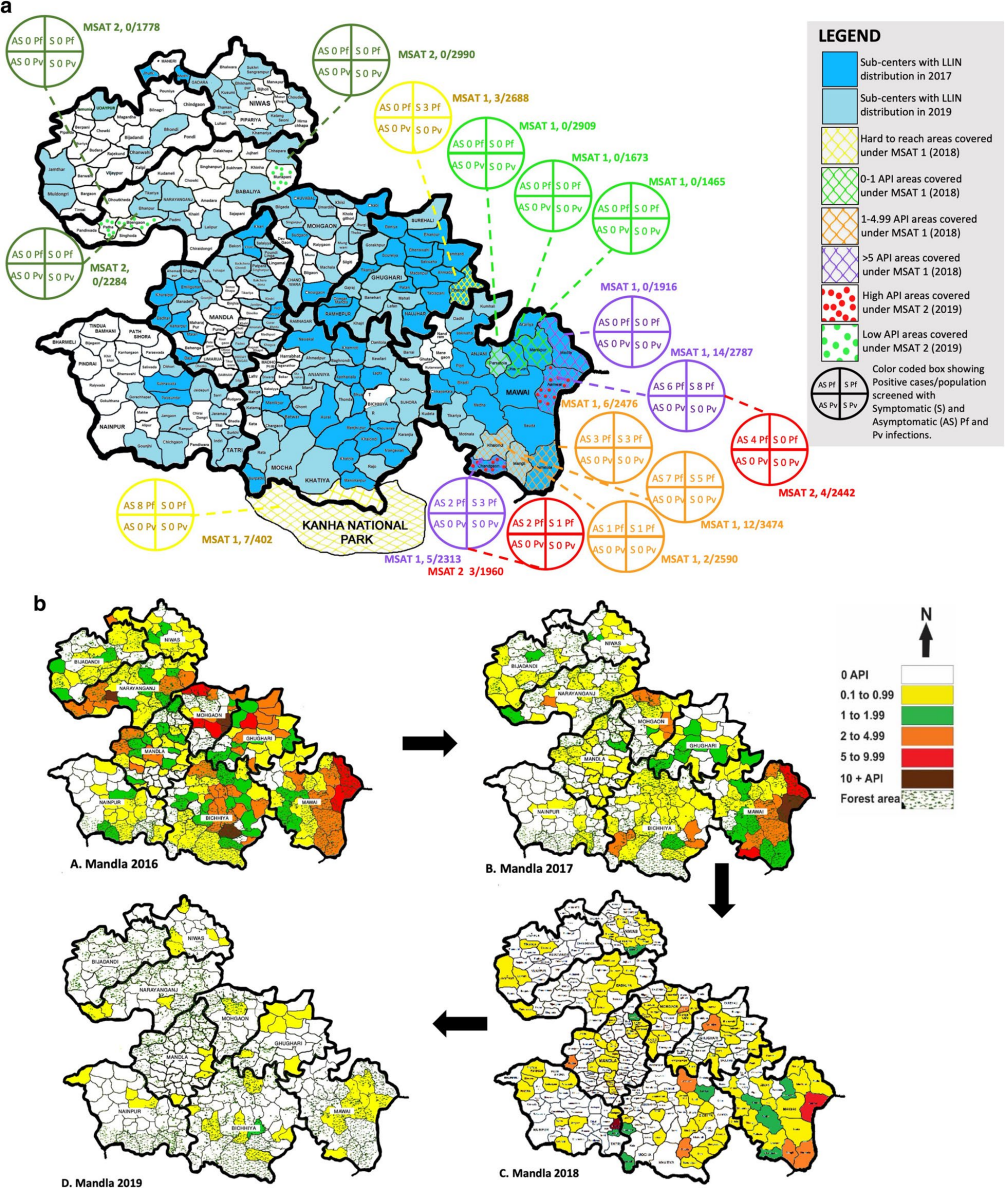
**a** Three mass screening and treatment (MSAT) exercises from September 2017 to February 2020. First MSAT was conducted in September to November 2018 in hard to reach areas, areas with API 0-1, API 1-4.99, and API more than 5. Additional area of Kanha National Park which is inaccessible to routine surveillance was also included. Total 28,469 residents were tested and 50 positive cases were diagnosed, out of which 27 were asymptomatic in nature. Second MSAT was conducted in June 2019 in two areas–high API and low API. Total 11,454 residents were tested and seven positive cases were diagnosed, out of which six were asymptomatic in nature. Third MSAT was conducted in December 2019 as a ring around the cryptic cases in 50 households. Total 8467 residents were tested and three cases were found, all asymptomatic in nature. **b** Map of Mandla showing malaria prevalence (API) from 2016 to 2019

MSAT rounds were conducted to identify extent of asymptomatic infection reservoirs in the study areas. In the first round, with the 74% (28,527/38,248) coverage of the target population, we detected 50 *P. falciparum* cases (0.18%) as compared to 0.06% prevalence through regular surveillance during the same time-period. These cases were found in areas with moderate to high API. Amongst the positive cases, slightly more than half (54%) were asymptomatic, while the rest were having malaria-like-symptoms (fever, headache, vomiting, chills) (Fig. 4a).

In the second round of MSAT, which was conducted in the low and high API areas, covering 77% (11,363/14,700) of the target population, seven *P. falciparum* cases were detected (0.06% prevalence) from high prevalence areas. This compared to 0.03% prevalence through the regular surveillance during the same time-period. Out of these seven cases, six were asymptomatic (Fig. 4a).

The third round of MSAT was conducted as a ring around the cryptic cases in 50 households. If any positive case was diagnosed during the screening, the entire village was covered under MSAT. A coverage of 82.6% (8467/10,246) of the target population was reached and three positive cases (0.03% prevalence) were detected from low prevalence areas of Niwas and Bijadandi blocks.

The decline of Annual Parasitic Incidence (API) from 2016 to 2019 is shown in Fig. 4b. In 2016, when the operational plan was being developed, the data from Government of MP revealed that a total of 77 sub-centres were free from malaria. Out of the total 297 sub-centres, in 2017, 143 sub-centres were free of malaria, which increased to 198 in 2018, and 211 in 2019 (Fig. 4b). Out of these sub-centres, few sub-centres were identified with greater than five API (malaria hotspots). These hotspots were targeted using different vector control interventions over the course of 24 months with continuous active surveillance and case management. These eight hotspots in 2016 reduced to three in 2017, further to two in 2018, and dropped to zero in 2019 (Table 4).

**Table 4:** Based on the API data, there were eight hotspots in the year 2016

Block	SCs	2016	VCI	AS/CM	2017^a^	VCI	AS/CM	2018^a^	VCI	AS/CM	2019^a^	VCI	AS/CM
Nainpur	Bhadiya	0.00	None	+	0.00	None	+	11.39	LLIN	+	1.34	IRS	+
Mohgaon	Munu	9.46	IRS	+	0.00	None	+	0.00	None	+	0.00	None	+
	Mungwani	8.45	IRS	+	2.77	IRS	+	0.46	None	+	1.03	LLIN	+
	Silgiti	34.95	IRS	+	2.05	IRS	+	0.00	None	+	0.00	None	+
	Umardeeh	5.57	IRS	+	2.49	IRS	+	0.82	None	+	0.00	None	+
	Chubhawal	8.55	IRS	+	2.04	IRS	+	0.00	None	+	0.00	None	+
Bicchiya	Khatola	16.65	IRS	+	1.51	IRS	+	2.68	LLIN	+	0.00	None	+
Mawai	Amwar	7.83	IRS	+	10.01	LLIN	+	6.42	LLIN	+	0.99	None	+
	Madfa	3.14	IRS	+	7.15	LLIN	+	0.37	None	+	0.00	None	+
	Chandgaon	0.94	None	+	9.52	LLIN	+	3.63	LLIN	+	1.37	LLIN	+
Narayanganj	Bhaanpur	12.11	IRS	+	2.18	IRS	+	0.00	None	+	0.00	None	+
Total scs		8			3			2			0		

All these hotspots were covered under IRS scheme. In 2017, after operationalization of MEDP, these hotspots were reduced to 3 owing to robust surveillance and case management along with LLIN distribution. In the year 2018, from the same cohort, only one hotspot remained along with appearance of a new hotspot in Nainpur block due to mass influx of imported cases in the region. With successful implementation of T4, monitoring and tracking of migratory population, monitoring of LLIN usage, supervision of IRS, and capacity building; MEDP was able to resolve all the hotspots in the year 2019

*VCM* Vector Control Interventions

*AS/CM* Active Surveillance and Case management

^a^In 2017, LLINs distributed in SCs with API more than 5; In 2019, LLINs distributed in SCs with API more than 1.In 2017, IRS done in SCs between API 1 and 4.99; In 2018, IRS done in SCs between API 1 and 4.99; In 2019, IRS done in areas where LLIN was not distributed

### Passive surveillance

In the District Hospital and Rotary health camp studies, a total of 503 and 509 fever cases were tested, respectively. No positive malaria case from these facilities and community-based studies were detected. These two sub-studies provided a random sample from all over Mandla district, and an independent test of the impact of work in this district. Private practitioners were also covered under the sentinel surveillance network for the collection of passive data on malaria cases. It was revealed that almost half of the total patient who visited these practitioners had the chief complaint as ‘Fever’. A total of 0.13% (11/8416) were found positive for the malaria parasite (Table 5).

**Table 5:** Data from Sentinel Surveillance System (SSS)

Categories	Total sentinel sites	No. of active practitioners	Practitioners who provided data	OPD numbers (April 2019 to May 2020)	Fever cases	Tested for malaria	Malaria positive
Category 1–Registered allopathic practitioners (MBBS)	7 (Mawai 1, Nainpur 1, Mandla 5)	22	7	1449	965	651	4
Category 2–Registered AYUSH practitioners (BAMS, BHMS, BUMS)	46 (Bicchiya 13, Bijadandi 6, Ghughari 4, Mandla 10, Mawai 2, Nainpur 8, Narayanganj 3)	55	41	43002	20475	7765	7
Category 3–Degree holders (para-medical, non-medical, lab technicians etc.)	13 (Mandla 2, Mawai 4, Niwas 7)	90	0	N.A.	N.A.	N.A.	N.A.
Category 4–No formal qualification	29 (Mandla 2, Mawai 19, Mohgaon 8)	276	0	N.A.	N.A.	N.A.	N.A.
Total	95	443	48	44451	21440	8416	11

Only Category 1 and 2 have reported malaria cases. MEDP has ensured that all positive malaria cases have completed their radical treatment. (N.A. means Not Available)

## Discussion

Malaria elimination is a global public health objective. The first countries to be certified malaria-free by WHO were Grenada, Saint Lucia in 1962, followed by Hungary and Spain in 1964. USA and Canada were certified malaria free in 1970 and 1965, respectively [26]. The latest certifications were awarded to Algeria and Argentina in 2019 [26]. In the South-East Asian countries, Maldives and Sri Lanka have achieved the malaria-free status in 2015 and 2016, respectively [26]. Bhutan and China are on their way to be declared malaria-free [27, 28].

The Government of India along with other heads of the state pledged for a malaria-free nation by the year 2030 [29] and developed a road map for malaria elimination [30]. In this road map, the national agency for vector borne diseases, the National Vector Borne Disease Control Programme has classified the country’s states and districts into low, moderate and high, as per the Annual Parasitic Incidence. It is important to note that the National Framework for Malaria Elimination 2016–30 has defined the district as the unit of planning and implementation of the malaria elimination programme [2].

In support of the national objective to eliminate malaria, a public–private-partnership was initiated to demonstrate that malaria can be eliminated. The Mandla district of Madhya Pradesh was chosen for this project, which is known for tribal, rural and forest malaria, intra/interstate border malaria, and migratory malaria. In addition, the Mandla district offered varied geography and topography consisting of a national park, water reservoirs, dense forests, and hilly areas. The project has demonstrated about 91% decline in the indigenous malaria cases in the district’s nine blocks (1233 villages), with 90% decline in three high prevalence blocks. Out of 1233 villages, a total of 1096 villages reported zero indigenous malaria cases from June 2017 to May 2018. This number rose to 1173 in 2018–19, and to 1183 in 2019–20, demonstrating expansion of malaria elimination net in the district.

The malaria elimination strategies included combination of active surveillance and case management focusing on early diagnosis and prompt treatment [15], robust training and monitoring of field staff, mapping of entire population and use of the indigenously developed mobile app based tool for surveillance, case management, human resource and supply chain management, regular supervision and monitoring of vector control interventions along with entomological studies. This combination approach in malaria elimination protocols yielded an impressive 91% reduction of cases in 3 years. The key to this level of reduction of cases was operational, management and technical controls implemented through weekly reviews.

A 80% reduction was also achieved in the state of Odisha using the CCMP and DAMaN approach [7] and several other studies have also demonstrated malaria control in India [31, 32]. All these studies used one or more strategies for malaria control viz. early diagnosis and complete treatment, vector control using LLIN/IRS/Larvivorous fishes, IEC/BCC, Mass Screening and Treatment (MSAT). Evidence shows that strengthening surveillance may control the malaria leading to elimination [8, 33–35].

Using robust surveillance, more than 80% of febrile cases were captured within 5-days of self-reported fever and provided diagnosis and treated for malaria. It should be noted that the Village Malaria Workers (VMWs) did not measure the temperature, because the National Guidelines state that every individual who either has fever or history of fever within a fortnight or malaria like symptoms should be tested using a RDT or a blood slide [30].

The use of electronic real-time data collection and reporting helped in prompt response backed with high quality evidence. The project has found more cases of malaria in adults, which is contrary to the observation of highest burden in children in Africa [1]. During the period of three years, only 1.3% of the individuals reported fever more than once, which is another indicator of reduction of malaria transmission in this district. Further, the mean time lag between two episodes of fever was more that 60 days, which also ruled out the effect of persistent HRP2 strains of *P. falciparum* parasites in the community as the project used RDT as the diagnostic tool.

The three rounds of MSAT revealed that most cases were found from the high to moderate API or hard-to-reach areas and only three cases from low endemic areas. This implies that asymptomatic cases reside predominantly where malaria transmission is present. Another important observation was drastic reduction of cases found over three cycles of MSAT, from 0.18% in Sep-Oct 2018 to 0.06% in June 2019 to 0.03% in December 2019. These observations demonstrate that the MSAT along with combination of case management and vector control were significantly reducing the burden of malaria at population level.

Therefore, in areas with moderate to high endemicity, it would be useful to conduct targeted MSATs in addition to active surveillance for achieving malaria elimination goals [36, 37]. For low endemic areas, asymptomatic cases were found around the cryptic cases indicating the need for investigations around cases in communities. The present findings and conclusions are in agreement with the observations of MSAT done in a high transmission area of Ghana [38, 39].

The sub-centres with API more than five were classified as the malaria hotspots. These areas appeared to maintain malaria transmission in low transmission season and act as reservoirs for transmission during the high transmission season. At the initiation of the study in 2016, eight hotspots were identified and targeted with tailored interventions. Within a timespan of 2 years, the project team was able to clean all the hotspots from the entire district. Similar strategy and suggestion have been proposed previously [39, 40]. Therefore, heterogeneity in malaria transmission within the district needs to be identified and targeted to achieve elimination.

The tracking of origin of introduced cases is important as a significant number of malaria cases were introduced into Mandla from a distant state of Telangana. After encountering the first cases in April 2018, tracking of migratory population was started in the Mandla district. This was done by maintaining an ‘active migration register’ which recorded the data of departure and expected date of arrival of the migratory workers. Upon arrival, these individuals were promptly tracked in their respective villages using the live household data from the SOCH mobile application and tested. During mass arrivals, they were intercepted at the bus stops and all febrile cases were tested. In some cases, up-to 25 patients were diagnosed in a bus carrying 30 passengers from malaria-endemic regions outside Mandla district. Therefore, the monitoring and vigilance of intra/inter district/state is necessary to achieve the elimination goal, which has been highlighted before [40]. For this to be effectively done, it would be useful to manage working relationship with labour contractors, so that a uniform and verifiable system of testing and treating can be implemented.

The vector control strategies of LLIN and IRS are used for interrupting the mosquito to human contact and serve as an important component of malaria control and elimination. Studies from India, Ethiopia Mozambique, Senegal have also demonstrated that IRS along with LLIN has reduced malaria transmission [31, 41–43]. As part of MEDP, the effect of direct observation-based monitoring and evaluation of these vector control measures in reducing and sustaining the malaria case load was observed. Similar advisory was advocated by Myanmar as a strategy for malaria elimination [44]. The facility-based study of malaria in the District Hospital and community-based study in the Rotary’s medical camp revealed zero malaria positive cases, which provided secondary validation of reduction of malaria cases all over Mandla district.

## Conclusion

The results of this study provide confidence in suggesting that malaria elimination in India may be achievable within a stipulated timeline. A significant reduction of malaria cases by about 91 percent was achieved in 33 months of intervention in which 1183 out of 1233 villages (96%) reported zero indigenous malaria cases from June 2019 to May 2020. Using active surveillance, imported malaria cases were identified and treated, which prevented outbreaks of malaria. The results of MSAT showed significance of its utility in moderate and high transmission areas. This project has revealed that monitored utilization of resources increases the quality of services that are needed for malaria elimination. Finally, the success of present project relied on the management, operational and technical controls combined with weekly reviews.

## Data Availability

We have reported all the findings in this manuscript. The hardcopy data is stored at MEDP Office in Mandla, Madhya Pradesh and National Institute of Research in Tribal Health (NIRTH), Indian Council of Medical Research (ICMR), Jabalpur, Madhya Pradesh. Softcopy data is available on the project server of MEDP hosted by Microsoft Azure. If anyone wants to review or use the data, they should contact: Dr. Altaf A. Lal Project Director–Malaria Elimination Demonstration Project, Mandla. Foundation for Disease Elimination and Control of India, Mumbai, India 482,003. E mail: altaf.lal@sunpharma.com

## Abbreviations

API: Annual parasitic incidence
ATP: Advance tour plan
CCA: Cluster combination approach
CCMP: Comprehensive case management programme
DAMaN: *Durgam Anchalare* Malaria *Nirakarana*
FDEC: Foundation for disease elimination and control of India
GoMP: Government of Madhya Pradesh
ICMR: Indian Council of Medical Research
IEC: Institutional Ethical Committee
LLIN: Long-lasting insecticide-treated nets
MEDP: Malaria elimination demonstration project
MFC: Malaria field coordinator
MSAT: Mass screening and treatment
NIRTH: National Institute of Research in Tribal Health
NSP: National strategic plan
NVBDCP: National vector borne disease control programme
OPD: Out Patient Department
SSS: Sentinel surveillance strategy
T4: Track, Test, Treat, and Track
VMW: Village malaria worker
WHO: World Health Organization
